# Predicting geographic distribution and habitat suitability of *Opuntia streptacantha* in paleoclimatic, current, and future scenarios in Mexico

**DOI:** 10.1002/ece3.10050

**Published:** 2023-05-01

**Authors:** Israel Cruz‐Jiménez, Pablo Delgado‐Sánchez, María de la Luz Guerrero‐González, Raúl Puente‐Martínez, Joel Flores, José Arturo De‐Nova

**Affiliations:** ^1^ Facultad de Agronomía y Veterinaria Universidad Autonóma de San Luis Potosí San Luis Potosí Mexico; ^2^ Desert Botanical Garden Phoenix Arizona USA; ^3^ División de Ciencias Ambientales Instituto Potosino de Investigación Científica y Tecnológica San Luis Potosí Mexico

**Keywords:** arid and semiarid zones, Chihuahuan desert, distribution modeling, ecological modeling, optimal habitat, paleoclimatic period, potential distribution

## Abstract

Mexican territory is one of the centers of origin and dispersion of the genus *Opuntia*, where several of its species have been an important plant resource for people in arid and semiarid zones. *Opuntia streptacantha* is widely distributed in Mexico; however, precise aspects of its geographic distribution and ecological status are still unknown. Here, we modeled its potential distribution under paleoclimatic, current, and future conditions through maximum entropy and predictions from 824 records and seven environmental variables. Potential distribution of *O. streptacantha* in the interglacial period was contracted and slightly north than current distribution, with 44,773 km^2^ of optimal habitat. In other past periods, the central location of potential distribution coincides with the actual current distribution, but the period of the last glacial maximum was characterized by 201 km^2^ of very suitable habitat, absent in interglacial, current, and future periods. The future model suggests that potential distribution will move toward the south of the Mexican territory. *Synthesis and applications*. The potential distribution of *O. streptacantha* can be applied for the conservation and management of the species, and also in the selection of areas with crassicaule scrubs for protection, conservation, and reproduction of species resistant to the hostile conditions of arid and semiarid zones of Mexican territory, where the structure and composition of the vegetation will be affected in the next 100 years.

## Graphical abstract

Potential distribution under paleoclimatic, current, and future conditions through maximum entropy and predictions was modeled for *Opuntia streptacantha*. In the interglacial period, its distribution was contracted and slightly north than current, with 42,850 km^2^ of optimal habitat including 27 km^2^ of very suitable habitat.

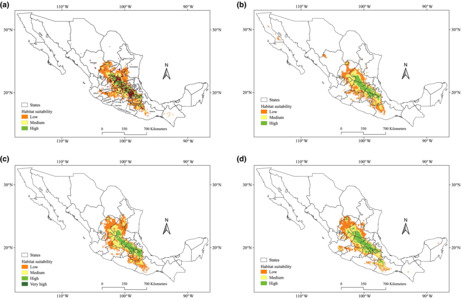

## 1 INTRODUCTION

The projected climate changes in the last 100 years, such as global warming, are comparable in magnitude to the changes in the last 65 million years (Diffenbaugh & Field, 2013; Kemp et al., 2015) and are undoubtedly affecting the distribution of species worldwide. However, it remains uncertain how ecosystem structure will be affected (Berg et al., 2010). To mitigate the effects of climate change on arid and semiarid ecosystems, we need to develop efficient conservation strategies by modeling the distributions of representative species in these ecosystems to identify regions where environmentally sensitive species exist or are likely to exist (Qin et al., 2017; Staudinger et al., 2012).

Geographic distribution models are relevant for ecological and biological conservation applications (Peterson et al., 2011; Phillips & Dudík, 2008) and allow to predict suitable environmental conditions for species as a function of environmental variables (Phillips et al., 2006). Ecological niche models, based on geographical records, are used to infer potential distribution pattern for a given species (Ibarra‐Díaz et al., 2016; Pérez‐García & Liria, 2013). This help to define the fundamental niche, which indicates the multivariate range of physiological tolerances to climatic variables, in which a given species will have positive growth rates (Soberón & Peterson, 2011).

Currently, the most used models for predicting species distribution are bioclimatic modeling (BIOCLIM) (Busby, 1991), domain environmental envelope (DOMAIN) (Carpenter et al., 1993), ecological niche factor analysis (ENFA) (Hirzel & Guisan, 2002), generalized additive model (GAM) (Guisan et al., 2006), genetic algorithm for rule set production (GARP) (Stockwell, 1999), and maximum entropy (MaxEnt) (Phillips et al., 2006). These models are based on the concept of ecological niche, relating biological information with environmental information, and subsequently identifying areas where there are no previous records of the species, thus obtaining the species' distribution area (Peterson et al., 2011). Among these models, the MaxEnt model has been widely used, because it works well with incomplete data or species presence‐only data (Phillips et al., 2006). MaxEnt is based on the principle of maximum entropy, a machine learning technique that uses a species' presence and background environmental data (Pearson et al., 2007), with the restriction that the expected value for each environmental variable in the distribution of a species must agree with its empirical average (Baldwin, 2009; Phillips et al., 2004, 2006). The objective of this approach was to predict which areas of the region satisfy the ecological niche requirements of the species and are therefore part of its potential distribution (Anderson & Martínez‐Meyer, 2013). This could be particularly relevant to design conservation strategies based on knowing the conditions where the survival of the species is suitable (Phillips et al., 2004), considering the future effects of climate change.

The family Cactaceae Juss. is the second largest neotropical family of quasi‐endemic angiosperms (Hunt et al., 2006), represented by succulent plants with specialized life forms (Gibson & Nobel, 1986). They are an important floristic component of the arid and semiarid zones of America. This family includes between 1500 and 1800 species, most of them distributed in Mexico (Anderson, 2001; Majure et al., 2012). They are plants with morphophysiological adaptations that allow them to develop in arid environments (Nobel, 1996). One of the largest genera in Cactaceae is the genus *Opuntia*, a native lineage of Mexico, where it was originated and diversified (Barthlott & Hunt, 1993; Bravo‐Hollis, 1978; García‐Zambrano et al., 2009).

*Opuntia streptacantha* Lem. is a dominant and massive key species (3–6 m height) of the southern Chihuahuan Desert crassicaule scrubs (Janzen, 1986; Miranda & Hernández‐Xolocotzi, 1963; Vargas‐Mendoza & González‐Espinosa, 1992). It is a highly branched arborescent species with a well‐defined trunk. Its cladodes have been used as food and forage for different cultures in Mexico. It is commonly known as “nopal cardón,” “tuna cardona,” and “nopal hartón” (Bravo‐Hollis, 1978; Scheinvar et al., 2008). It occurs on hillsides as part of the crassicaule scrubs and its populations form distinctive patches of vegetation called “nopaleras,” up to 500 m^2^ and surrounded by bare ground, as the vegetation decreases in density and height uphill (Bravo‐Hollis, 1978; Scheinvar et al., 2008; Yeaton & Cody, 1979; Yeaton & Romero‐Manzanares, 1986).

In this study, we modeled the potential distribution of *O. streptacantha*. Our goals were to identify environmental variables that are correlated with the *O*. *streptacantha* range, to predict the past, current, and future distribution, and to analyze the habitat suitability and effects of climate change on the species range. Our question was how the distribution of the species was in the past and how it could change in future scenarios. This information will impact the protection, conservation, and recovery of wild populations of this key species and their plant communities, which is also relevant for the conservation of the Chihuahuan Desert crassicaule scrubs.

## 2 MATERIALS AND METHODS

### 2.1 Database

**FIGURE 1:**
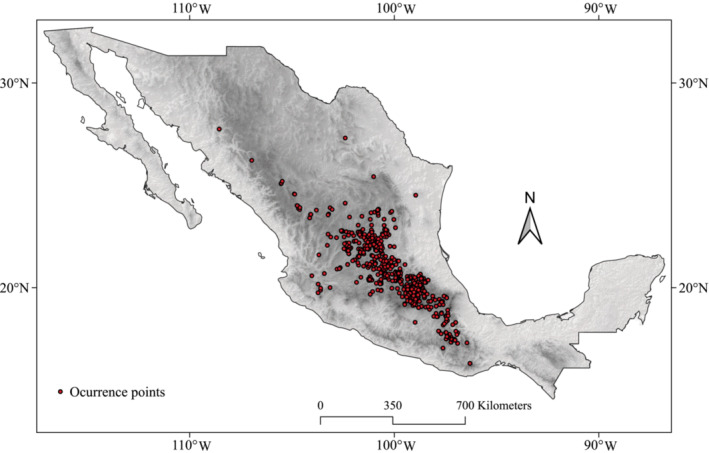
Distribution of the 824 records of *Opuntia streptacantha* used in this study.

Our database includes all the available geographic information from distribution records of *O. streptacantha* in Mexico. Occurrence data for the species were extracted from the Gbif platform (GBIF, 2020) and databases from Herbario Nacional de México (IBUNAM), Herbario Isidro Palacios (SLPM), and the Red de Herbarios del Noreste de México (RHNM, 2020) that includes the herbaria of Universidad Autónoma de Baja California (BCMEX), Centro de Investigaciones Biológicas del Noroeste (HCIB), Universidad de Sonora (USON), Universidad Autónoma de Sinaloa (UAS), Herbario Regional CIAD‐Mazatlán (HCIAD), and Centro Interdisciplinario de Investigación para el Desarrollo Integral Regional Unidad Durango (CIIDIR). A depuration of the database was conducted, including only those records with geographic location data. A total of 824 records (Figure 1) from years 1970 to 2020, representing the natural distribution recognized for *O*. *streptacantha*, were included in the analyses, and are available via ZENODO https://doi.org/10.5281/zenodo.7796359.

### 2.2 Environmental variables

Species distribution is an ecological process affected by temperature, precipitation, and geographic barriers such as mountain chains (Adams & Burg, 2015; Qin et al., 2017). Nineteen bioclimatic variables were included in our model to determine the potential distribution of *O. streptacantha*, with a resolution of 30″ (ca. 1 km at the ground level), from the WorldClim dataset version 1.4 (Hijmans et al., 2005; Otto‐Bliesner et al., 2008) for past scenarios and the version 2.1 (Fick & Hijmans, 2017) for current and future scenarios (http://www.worldclim.org). Current climate data include averages for the years 1970–2000. Paleoclimatic data, including the Last Interglacial (LIG; ~120,000–140,000 years BP) and Last Glacial Maximum (LGM; ~22,000 years BP), are derived from Community Climate System Model version 4 (CCSM4) and Model for Interdisciplinary Research on Climate version 6 (MIROC) (Wang et al., 2018). These periods were relevant for distribution changes, demographic dynamics, and genetic patterns in several species in Neotropics and particularly in the Chihuahuan desert (Castellanos‐Morales et al., 2016; Ramírez‐Barahona & Eguiarte, 2013; Scheinvar et al., 2017). Future climate data are derived from three Shared Socioeconomic Pathways (SSPs) 585 for the years 2050 (mean of predictions for years 2041–2060), 2070 (mean of predictions for years 2061–2080), and 2090 (mean of predictions for years 2081–2100). These are the most recent projections of General Circulation Models (GCMs) evaluated by the Coupled Model Intercomparison Project Phase 6 (CMIP6) at the 18th session of the Working Group on Coupled Modelling (EGCM) (Eyring et al., 2016). The data obtained were first clipped for the surface of Mexico and then converted to ASCII file format using QGIS 3.14 (QGIS, 2020).

To avoid multicollinearity among the bioclimatic variables, highly correlated variables (*r* ≥ .85 Pearson's correlation coefficient) were eliminated from further analyses (Graham, 2003). Seven bioclimatic variables were used to model the distribution: diurnal temperature oscillation (Bio2), isothermality (Bio3), temperature seasonality (Bio4), annual temperature oscillation (Bio7), precipitation seasonality (Bio15), precipitation of warmest quarter (Bio18), and precipitation of coldest quarter (Bio19). These retained variables have ecological relevance implied in key physiological processes (e.g., photosynthesis and water uptake) that are directly affected by air temperature and precipitation (Austin, 2002; Low et al., 2021).

### 2.3 Species distribution modeling

The potential distribution of *O. streptacantha* in different climatic scenarios was predicted using a maximum entropy algorithm in MaxEnt version 3.4.4 (Phillips et al., 2004, 2006). This program uses presence‐only data to predict the distribution of a species based on the maximum entropy principle by estimating the distribution over geographical space. MaxEnt attempts to estimate a probability distribution of species presence that is as close to uniform as possible, but still subject to environmental conditions, and the resulting model is the quantification of habitat suitability for the species (Elith et al., 2011). We used “replicates” option with cross‐validation to estimate model capacity, with 75% of the geographical data selected for model training and 25% for model testing (Liu et al., 2005; Morueta‐Holme et al., 2010; Phillips & Dudík, 2008). The number of training repetitions was set to 10 to reduce uncertainty caused by outliers in bioclimatic variables associated with randomly selected training points. The maximum number of background points was set to 10,000 random points in Mexico. During initial model building, the percentage contribution of each bioclimatic variable was detected using the Jackknife test and variables with a low percentage contribution (<1%) (Deng et al., 2022) were eliminated. Subsequently, the MaxEnt model was refitted using six highly contributing bioclimatic variables with the data on the presence of *O. streptacantha* and modeling of the distribution of the species in the past, current, and future climate scenarios were performed. A threshold‐independent receiver operating characteristic (ROC) analysis was carried out to evaluate the performance of MaxEnt. This curve originates the area under receiver‐operating characteristic curve (AUC) statistic. The area under receiver‐operating characteristic curve values range from 0.5 (random) to 1.0 (perfect discrimination), where 0.5 to 0.7 model reliability is low, 0.7 to 0.9 signals a useful application of the model, and values above 0.9 are high reliability (Peterson et al., 2008; Swets, 1988). Jackknife results and response curves were used to evaluate the importance of each environmental variable for species distribution. The results of MaxEnt models were visualized in QGIS 3.14 (QGIS, 2020). Based on the classification proposed by Zhang et al. (2019), four classes of potential habitats were reclassified: no potential (<0.2), low (0.2–0.4), medium (0.4–0.6), high (0.6–0.8), and very high (0.8–1.0). In each model, the optimal distribution area was calculated and classified as high or very high suitability habitat (0.6–1.0).

## 3 RESULTS

### 3.1 Precision and dominant environmental factors in the current scenario model

**FIGURE 2:**
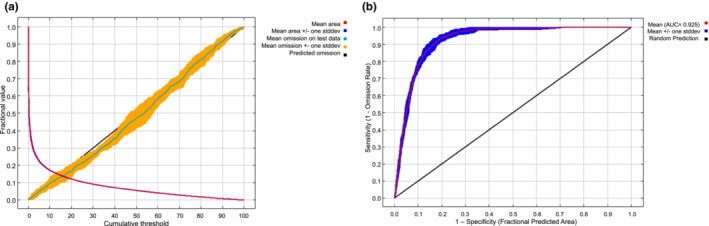
Predictive effect of the MaxEnt model of *Opuntia streptacantha*. (a) Omission curve and predicted area. (b) Receiver operating characteristic curve of potential distribution prediction.

**FIGURE 3:**
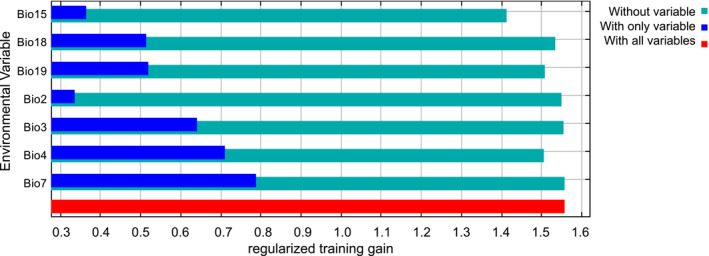
The Jackknife test to evaluate the relative importance of environmental variables for *Opuntia streptacantha* in Mexico. Bio15 (precipitation seasonality), Bio18 (precipitation of warmest quarter), Bio19 (precipitation of coldest quarter), Bio2 (diurnal temperature oscillation), Bio3 (isothermality), Bio4 (temperature seasonality), and Bio7 (annual temperature oscillation). The blue bars indicate the gain using the single environmental variable, the green bars indicate the gain excluding the single variable from the full model and the red bar indicates the gain considering all variables.

The distribution model for *O. streptacantha* generated with MaxEnt for the current scenario showed a prediction omission rate with high agreement with the omission rate of the test sample (Figure 2a). The ROC curve displayed an AUC value above the random prediction (0.931), with high sensitivity and reliability (Figure 2b). Temperature seasonality (Bio4) contributed most of the model with 42.8%, followed by precipitation of the coldest quarter (Bio19) with 15.6%, precipitation seasonality (Bio15) with 13.1%, precipitation of the warmest quarter (Bio18) with 12.4%, diurnal temperature oscillation (Bio2) with 10.3%, and annual temperature oscillation (Bio7) with 5.5%. The cumulative contribution of these six factors was 99.7%. The Jackknife test indicates that variables that most contribute to the persistence and geographic distribution of the species were Bio7, Bio4, Bio3, Bio19, and Bio18 (Figure 3), with Bio7 being the most important when used alone.

**FIGURE 4:**
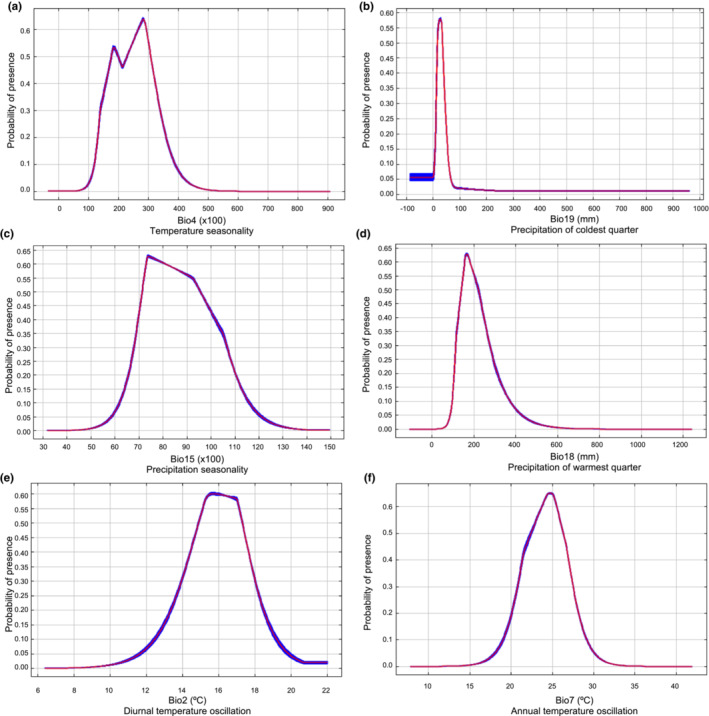
Response curves of the six most important environmental factors in modeling habitat distribution for *Opuntia streptacantha*.

MaxEnt results for the current scenario (years 1970–2000) indicate that the occurrence of *O. streptacantha*, according to response curves thresholds (probability of presence of the species >0.6), increases with relatively low temperature seasonality values (Bio4) (Figure 4a) with the optimum range being between 26.5 and 29.5°C. For the precipitation of the coldest quarter (Bio19) ranging between 18.75 and 31.25 mm (Figure 4b) and the seasonality of precipitation (Bio15) between 72.5 and 80 mm (Figure 4c), the probability of occurrence was greater than 60%. Precipitation of the warmest quarter (Bio18) ranges between 155 and 181.3 mm (Figure 4d). Finally, diurnal temperature oscillation (Bio2) and annual temperature oscillation (Bio7) also determined the occurrence of the species, ranging between 15.3–16.25°C and 24.4–25.3°C as optimal habitats, respectively (Figure 4e,f).

### 3.2 Predicted current potential distribution

**FIGURE 5:**
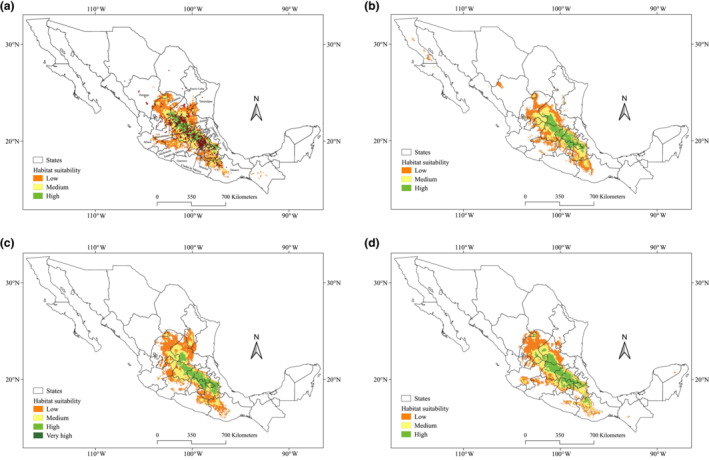
Predicted current and historical period habitat for *Opuntia streptacantha*. (a) Current suitable habitats with the occurrence of the species; (b) paleoclimate predictions for the last interglacial; (c) paleoclimate predictions from CCSM4 climatic model for the last glacial maximum (LGM); (d) paleoclimate predictions from MIROC climatic model for the LGM.

Suitable habitats for *O. streptacantha* at present were predicted in central Mexico (Figure 5a), including southeastern Zacatecas, central, eastern and western San Luis Potosí, eastern Jalisco, southeastern Tamaulipas, northern and eastern Guanajuato, central Querétaro, central and southern Hidalgo, northern and eastern Estado de México, northern Ciudad de México, Tlaxcala, central, eastern, and southern Puebla, and northwestern Oaxaca, where the species is known to be extant. Suitable habitat was also predicted in the state of Morelos, where the species is known to be absent. The area of the current potential distribution of *O*. *streptacantha* is significantly larger than the real occurrence in the Mexican territory. The model predicted 47,090 km^2^ of optimal habitat area, bounded to the north 22.89°, south 17.69°, east 98.65°, and west 100.62° (Table 1).

**TABLE 1:** Range and area of distribution of *Opuntia streptacantha* under different climate scenarios.

Period	Climate scenario	North	South	East	West	Area/km^2^
LIG		22.84°	18.78°	98.58	100.52°	44,773
LGM	CCSM4	22.83°	18.27°	99.12°	100.85°	50,175
	MIROC	22.74°	18.61°	99.13°	100.70°	59,847
Current		22.89°	17.69°	98.65°	100.62°	47,090
2050	MRI‐ESM2‐0	23.05°	17.70°	99.37°	100.58°	54,109
2070	MRI‐ESM2‐0	22.74°	17.80°	99.37°	100.56°	51,712
2090	MRI‐ESM2‐0	23.03°	16.86°	99.37°	100.53°	54,010

### 3.3 Predicted paleoclimate potential distribution

Paleoclimate (LIG) predictions reveal a larger and continuous distribution area for *O. streptacantha* in central México, including eastern Zacatecas, western San Luis Potosí, Guanajuato, Querétaro, Hidalgo, Morelos, northern and eastern Estado de México, northern and eastern Ciudad de México, northern, eastern, and western Tlaxcala and western Puebla. The models indicate that the distribution of *O*. *streptacantha* during the LIG was further north of its current distribution (Table 1, Figure 5b). Moreover, the model showed 44,773 km^2^ of optimal habitat, 4.9% smaller than the area of optimal habitat in the current climate scenario.

Paleoclimatic predictions of the CCSM4 climate model for the last glacial maximum (LGM) showed a significant change in area extension with a large increase of 5402 km^2^ in the predicted distribution compared with the last interglacial period (LIG), but closer to the area and location predicted for the current climate scenario, including a great part of Tlaxcala and Puebla (Figure 5c). The MIROC model included eastern Durango, eastern Jalisco, eastern Michoacán, eastern Guerrero, and northwestern Oaxaca (Figure 5d). The CCSM4 model generated 50,175 km^2^ of optimal habitat, including 201 km^2^ of very highly suitable habitat in southern Hidalgo, while the MIROC model generated 59,847 km^2^ of optimal habitat (Table 1).

### 3.4 Predicted future potential distribution

**FIGURE 6:**
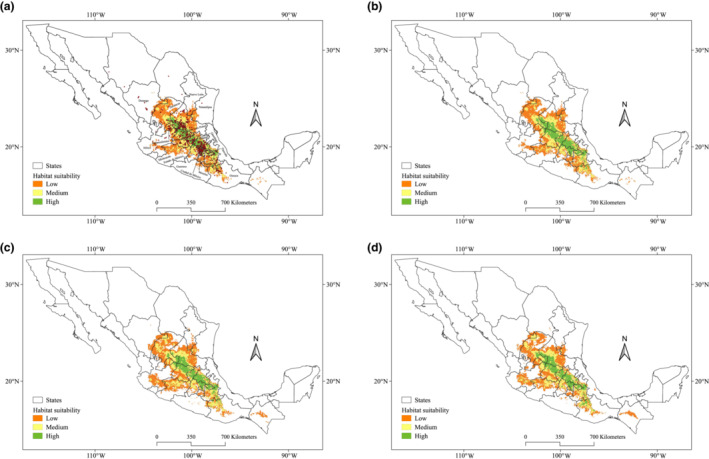
Predicted current and future period habitat for *Opuntia streptacantha*. (a) Current suitable habitats; (b) future period 2050; (c) future period 2070; (d) future period 2090.

The future predictions for the years 2050 (Figure 6b), 2070 (Figure 6c), and 2090 (Figure 6d) show a larger area of highly suitable habitat than the current climate and paleoclimatic scenarios, with 54,019 km^2^ (12.8% higher than current), 50,712 km^2^ (7.1% higher than current), and 52,138 km^2^ (10.2% higher than current), respectively (Table 1). Future predictions exhibit a maximum extension to the south at 16.94° for the year 2090, according to the predicted effects of climate change, including southwestern Zacatecas, northern Aguascalientes, central and southeastern San Luis Potosí, west Jalisco, north and west Guanajuato, central and south Queretaro, central and south Hidalgo, north, central and west Estado de México, north and west Ciudad de México, Tlaxcala, central and south of Puebla, and central and northeast of Oaxaca.

## 4 DISCUSSION

The global increasing average temperature threats the growth and survival of many wild species and their habitat (Brummitt & Bachman, 2010), as is the case of the *O. streptacantha*. Although tolerance to extreme temperatures has been reported for this genus (Nobel & De La Barrera, 2003), young individuals of *O. streptacantha* show a negative sensitivity to high and low temperatures that would endanger their survival (Ojeda‐Pérez et al., 2017), and the potential distributions of several wild species in Mexico will predictably change with global warming (Bellard et al., 2012; Contreras‐Medina et al., 2010; Gutiérrez & Trejo, 2014).

### 4.1 Environmental variables determining the distribution

The dominant variables that affected the distribution of *O. streptacantha* in the current scenario mainly involve seasonality and oscillation of temperature (Bio2, Bio4, Bio7) and precipitation (Bio15, Bio18, and Bio19). Ranges of most determining variables for optimal habitats like the seasonality of temperature (Bio15, 26.5–29.5°C), precipitation in the warmest quarter (Bio18, 155–181.3 mm), and precipitation in the coldest quarter (Bio19, 18.75–31.25 mm), are consistent with the current habitat requirements for the genus *Opuntia*, which grows well at 27–30°C and whose roots and stems are sensitive to temperature changes (Drennan & Nobel, 1998; Nobel & Bobich, 2002). These variables are relevant for photosynthesis and water uptake (Austin, 2002; Low et al., 2021). Variables related to rainfall (Bio15, Bio18, and Bio19) are relevant for *O. streptacantha* as a succulent plant that stores water in the rainy season and uses it efficiently during the dry season since the CAM activity (Delgado‐Sánchez et al., 2013). Studies have shown that *Opuntia* plants from 10 years of age generate a certain tolerance to low temperatures (2–6°C), thus ensuring their survival during the winter season in the arid and semiarid zones of Mexico (Nobel & De La Barrera, 2003). It was also reported that *O*. *streptacantha* seedlings exposed to constant temperatures of 4 or 40°C diminish photosynthetic activity and inhibit growth (Ojeda‐Pérez et al., 2017); even so, the plants of this species can tolerate drought by moving their chloroplasts (Delgado‐Sánchez et al., 2013). In addition, it has been described that the optimum temperature for seed germination of *Opuntia* species is 25°C (Bauk et al., 2017; Nobel, 2003; Rojas‐Aréchiga & Vázquez‐Yanes, 2000).

### 4.2 Climate scenarios

The potential distributions estimated for *O. streptacantha* in the past, current, and future scenarios, predicted by our models, are consistent with the expected effect of climate change on the species over time. Biodiversity around the world is vulnerable to the threats of climate change, which can compromise its distributional ranges (Bellard et al., 2012). The last 2.5 million years were times of intense environmental climatic changes, climatic cycles of growth, and the decline of the polar ice cap that occurred in North America, affecting global climatic conditions. Populations decreased in size, shrinking to so‐called Pleistocene refugia where they survived these difficult periods of climate change (Scheinvar et al., 2020). It has been argued that the climate of the LIG was warmest and wettest than the current, being the longest warm period (Wu et al., 2006, 2007); central Mexico was warmer and more humid in this period than today, with more precipitation in its coldest period (Metcalfe, 2006). The C_3_ plants dominated the past and only a small group of CAM plants existed (Chen et al., 2011). According to our results, *O*. *streptacantha* populations that survived the LIG could occupy areas where they found refuge from the warm and humid conditions. However, the climatic change during the LGM to coldest and driest conditions could produce a sudden demographic expansion in some species and the glacial descent of the mountains allowed seed‐mediated gene flow (Ornelas & Rodríguez‐Gómez, 2015). This could trigger the diversification and population dynamics in some cactus species in the Chihuahuan Desert and arid Poblano‐Oaxaca region, where *O*. *streptacantha* mainly occurs today. Similar patterns have been indicated for South American Cactaceae (Silva et al., 2018). The arid zones of Central and North America became drier after the last glacial maximum, leading to changes in the distribution of species (Aguirre‐Planter et al., 2020; Ramírez‐Barahona & Eguiarte, 2013). This explains a larger distribution of *O. streptacantha* in LGM than in LIG period, and similar distributions in LGM and the current, but with a larger optimal habitat today. The same pattern of contraction during the LIG period and expansion in the LGM period has been reported for other taxa adapted to arid ecosystems such as *Melampodium leucanthum* (Rebernig et al., 2010), *Astrophytum* (Vázquez‐Lobo et al., 2015), *Agave lechuguilla* (Scheinvar et al., 2017), and *Agave kerchovei* (Aguirre‐Planter et al., 2020), and agree with the idea of modernization of North American deserts during the dry intervals of the Quaternary period (De‐Nova et al., 2020; Graham, 2010). The CAM plants are efficient in use of water and in maintaining carbon gain in view of low extreme water stress (Eguiarte et al., 2013; Hernández‐Hernández et al., 2014; Reyes‐Agüero et al., 2006; Scheinvar et al., 2017).

Our results revealed a larger current potential area than the real distribution for *O. streptacantha* through northern and southern Mexico. The potential distribution of its optimal habitat is consistent with the distribution mentioned by Bravo‐Hollis (1978) and Scheinvar et al. (2008). This is related to the geological history of the territory, soils, and elevation, which reflect the diverse landscape with rich plant associations where the species occurs (Dávila et al., 2002; Morrone, 2019; Valiente‐Banuet et al., 2009). Individuals of *O. streptacantha* mainly grow on hillsides and slopes, where temperature, humidity, and fluctuate more than in lowlands. Climatic fluctuations have long been suggested as a key factor in cactus family diversification and distribution (Homer et al., 2020; Nyman et al., 2012), with temperature being one of the main determinants for distribution (Fitter & Hay, 2002).

The long dispersal distances of *O. streptacantha* in central Mexico has been argued to have benefited from migratory Pleistocene megafauna such as the woolly mammoth (Majure et al., 2012), as well as countless herbivores that make up the local fauna such as turtles, birds, rabbits, bats, and even coyotes (Mellink & Riojas‐López, 2002), promoting a more southern distribution in the present. According to our predicted future scenarios, the distribution of *O. streptacantha* could move toward the south of the Mexican territory, covering areas of the state of Oaxaca with high and medium suitability and Chiapas with low suitability. Our results must be considered in planning land use management, especially around the sites the species inhabits now and could inhabit in the future, since as a key species of crassicaule scrubs the future distribution of the species can be used to prioritize areas for the protection of this ecosystem. More research is needed to determine whether crassicaule scrubs in Mexico in the existing protected areas adequately cover suitable habitats for *O. streptacantha*. Our results could be used too for quantifying habitat distribution patterns for threatened and endangered plant and animal species in the Chihuahuan Desert crassicaule scrubs helping the conservation and restoration efforts in Mexico.

## 5 CONCLUSION

This research projected the potential distribution of *O. streptacantha* under paleoclimatic, current, and future scenarios; and determined the dominant environmental variables that affect changes in the species distribution. The potential distribution here modeled for *O. streptacantha* can be applied for conservation and management of the species, and the preservation of crassicaule scrubs where it dominates. The future potential distribution model here estimated could help to identify future habitats, where *O. streptacantha* is not currently present, which is useful in selecting priority areas for its propagation. This information can be used in planning land use management, to prioritize areas for the protection of arid ecosystems, both for government agencies, skateholders, and actors, such as the Comisión Nacional de Areas Naturales Protegidas (CONANP), ONGs, and people living in the region. All the maps generated in this study can be downloaded via ZENODO https://doi.org/10.5281/zenodo.7796359.

## AUTHOR CONTRIBUTIONS

**Israel Cruz‐Jiménez:** Conceptualization (equal); data curation (equal); formal analysis (equal); investigation (equal); methodology (equal); visualization (lead); writing – original draft (equal); writing – review and editing (equal). **Pablo Delgado‐Sánchez:** Conceptualization (equal); funding acquisition (lead); project administration (lead); resources (equal); software (equal); supervision (equal); validation (equal); visualization (equal); writing – original draft (equal); writing – review and editing (equal). **Maria de la Luz Guerrero‐González:** Conceptualization (equal); investigation (equal); writing – original draft (equal); writing – review and editing (equal). **Raul Puente‐Martínez:** Conceptualization (equal); investigation (equal); supervision (equal); writing – original draft (equal); writing – review and editing (equal). **Joel Flores:** Conceptualization (equal); investigation (equal); methodology (equal); supervision (equal); validation (equal); visualization (equal); writing – original draft (equal); writing – review and editing (equal). **José Arturo De‐Nova:** Conceptualization (equal); investigation (equal); methodology (equal); software (equal); supervision (equal); validation (equal); visualization (equal); writing – original draft (equal); writing – review and editing (equal).

## CONFLICT OF INTEREST STATEMENT

We declare no conflict of interest.

## Data Availability

Geographical data and maps are available via ZENODO https://doi.org/10.5281/zenodo.7796359.
